# Increased Absorption of Thyroxine in a Murine Model of Hypothyroidism Using Water/CO_2_ Nanobubbles

**DOI:** 10.3390/ijms25115827

**Published:** 2024-05-27

**Authors:** Maria Cecilia Opazo, Osvaldo Yañez, Valeria Márquez-Miranda, Johana Santos, Maximiliano Rojas, Ingrid Araya-Durán, Daniel Aguayo, Matías Leal, Yorley Duarte, Jorge Kohanoff, Fernando D. González-Nilo

**Affiliations:** 1 Facultad de Medicina Veterinaria y Agronomía, Instituto de Ciencias Naturales, Universidad de las Américas, Santiago 7500975, Chile; 2 Laboratorio de Endocrino Inmunología, Millenium Institute on Immunology and Immunotherapy, Facultad de Ciencias de la Vida, Universidad Andrés Bello, Santiago 8370146, Chile; johana.santos77@gmail.com; 3 Núcleo de Investigación en Data Science, Facultad de Ingeniería y Negocios, Universidad de las Américas, Santiago 7500975, Chile; oyanez@udla.cl; 4 Center for Bioinformatics and Integrative Biology (CBIB), Facultad de Ciencias de la Vida, Universidad Andres Bello, Santiago 8370146, Chilemaximiliano.rojas@unab.cl (M.R.);; 5 Departamento de Química Orgánica y Fisicoquímica, Facultad de Ciencias Químicas y Farmacéuticas, Universidad de Chile, Santiago 8380494, Chile; 6 Interdisciplinary Center for Neuroscience of Valparaíso, Faculty of Science, University of Valparaíso, Valparaíso 2340000, Chile; 7 Instituto de Fusión Nuclear “Guillermo Velarde”, Universidad Politécnica de Madrid, 28006 Madrid, Spain; 8 Atomistic Simulation Centre, Queen’s University Belfast, Belfast BT7 1NN, UK

**Keywords:** thyroxine, nanobubbles, drug delivery

## Abstract

Thyroxine (T4) is a drug extensively utilized for the treatment of hypothyroidism. However, the oral absorption of T4 presents certain limitations. This research investigates the efficacy of CO_2_ nanobubbles in water as a potential oral carrier for T4 administration to C57BL/6 hypothyroid mice. Following 18 h of fasting, the formulation was administered to the mice, demonstrating that the combination of CO_2_ nanobubbles and T4 enhanced the drug’s absorption in blood serum by approximately 40%. To comprehend this observation at a molecular level, we explored the interaction mechanism through which T4 engages with the CO_2_ nanobubbles, employing molecular simulations, semi-empirical quantum mechanics, and PMF calculations. Our simulations revealed a high affinity of T4 for the water–gas interface, driven by additive interactions between the hydrophobic region of T4 and the gas phase and electrostatic interactions of the polar groups of T4 with water at the water–gas interface. Concurrently, we observed that at the water–gas interface, the cluster of T4 formed in the water region disassembles, contributing to the drug’s bioavailability. Furthermore, we examined how the gas within the nanobubbles aids in facilitating the drug’s translocation through cell membranes. This research contributes to a deeper understanding of the role of CO_2_ nanobubbles in drug absorption and subsequent release into the bloodstream. The findings suggest that utilizing CO_2_ nanobubbles could enhance T4 bioavailability and cell permeability, leading to more efficient transport into cells. Additional research opens the possibility of employing lower concentrations of this class of drugs, thereby potentially reducing the associated side effects due to poor absorption.

## 1. Introduction

Current drug delivery systems often limit the effectiveness of medications due to poor bioavailability, unintended targeting, and potential side effects. To overcome these challenges and improve treatment outcomes, researchers are exploring novel drug carrier systems. Among these promising approaches, nanobubbles stand out for their unique properties. These nano-sized gas-filled particles offer several advantages, including high stability, customizable surface properties, and the ability to carry both hydrophilic and hydrophobic drugs. By acting as carriers or facilitators for therapeutic agents, nanobubbles have the potential to significantly improve drug delivery efficiency, target specific tissues or cells more precisely, and minimize unwanted side effects [1]. This work explores the use of nanobubbles as innovative drug delivery vehicles to improve T4 absorption in the context of a hypothyroidism murine model enhancing T4 bioavailability and cell permeability, leading to more efficient transport into cells.

### 1.1. CO_2_ Nanobubbles

Nanobubbles (NBs) are ultrafine bubbles, less than 200 nm in diameter, filled with gas that possess unique properties [2]. NBs are generated by gas injection (e.g., CO_2_, O_2_, air) through special porous filters or gas tubing [3] and are characterized by a remarkable longevity (metastability) in water [4] that can extend to several months. The application of NBs has increased vertiginously over the past years in several fields, particularly in biomedicine. CO_2_ nanobubbles hold significant importance in various fields due to their unique properties and potential applications. First, their small size and high surface-area-to-volume ratio make them excellent candidates for drug delivery systems [5]. Their small size allows for extravasation from blood vessels and site-specific release, while their stability and longer circulation time enhance drug delivery efficiency [6,7]. Several studies have explored the ability of nanobubbles to pass through cell membranes. Shin (2015) [8] provided insights into the growth dynamics and gas transport mechanism of nanobubbles. Recently, water vapor nanobubbles (VNBs) can be induced by external forces to release drugs and achieve therapeutic effects [9]. Here, VNBs were used for intracellular delivery, exploring potential long-term effects, limited in vivo applications, and emerging applications beyond intracellular delivery [10]. In particular, CO_2_ nanobubbles have been widely used in food and agriculture systems [2,11] but little is known about their potential therapeutic use as absorption promoters.

### 1.2. Thyroid Hormones

Thyroxine (T4) and triiodothyronine (T3) are secreted by the thyroid gland. T4 is secreted in a higher proportion than T3, being considered as the storage hormone [12]. T4 is synthesized in the thyroid gland through the oxidative coupling of two molecules of the amino acid tyrosine, which are appropriately iodinated in the 3 and 5 positions of the phenolic ring [13]. Thyroid hormones are vital for proper metabolism function and thyroid dysfunction can induce health problems [14]. Thyroid hormone deficiency is commonly known as hypothyroidism (Hypo) where both T4 and T3 levels are decreased and there is an increase in thyroid-stimulating hormone (TSH) levels in response to low levels of T4 [12]. Hypothyroidism is a worldwide problem, with a prevalence of 0.2% to 5.3% in Europe and 0.3% to 3.7% in the USA [15] depending on iodine intake and/or availability [16]. When diagnosed, hypothyroidism can be treated by daily administration of thyroxine [17]. An important issue that needs tackling is the difficulty of administering patients a stable dose, given the poor oral absorption of T4 [18,19]. Due to its hydrophobicity, drugs like T4 present low water solubility, decreasing their therapeutic effectiveness [20]. The average dosage for an adult under 50 is 1.7 µg/kg/day equivalent to 100–125 µg/day [21] for a typical adult, and it is mainly absorbed in the intestine, particularly in the duodenum, jejunum, and ileum [21], being directly influenced by the presence of food, coffee and by gastric pH decreasing bioavailability and delaying the time of maximum concentration, Tmax [22,23]. Moreover, highly prevalent conditions nowadays such as celiac disease, lactose intolerance, and *Helicobacter pylori* infection impair thyroxine absorption, and higher doses are required to treat those patients [23,24]. Current available commercial formulations range from tablets to intravenous solutions and more recently soft gel capsules and oral solutions. All of them have advantages and disadvantages that are related to the formulation and not to the thyroxine itself [21]. Despite seven decades of therapeutic use of thyroxine [25], a controlled-release method is still under development. 

In this work, we explore the ability of CO_2_-NBs to facilitate orally ingested T4 absorption using a transient hypothyroidism murine model and molecular dynamics (MD) simulation strategies to explain, at a molecular level, the possible mechanism underlying the facilitating role of CO_2_-NBs in T4 absorption in CO_2_-NB+T4-treated mice. Furthermore, in the present study, we elucidate a possible mechanism by which the interaction of nanobubbles with cell membranes facilitates the translocation of T4 across a cell membrane. The interaction of nanobubbles with cell membranes absorbs the gas available in their environment. For this reason, we present in this article a theoretical study of the T4 translocation process through a POPC lipid bilayer and another POPC membrane with CO_2_ molecules in its transmembrane region (POPC-CO_2_), resembling a cell membrane that has been treated with nanobubbles.

## 2. Results

**Figure 1:**
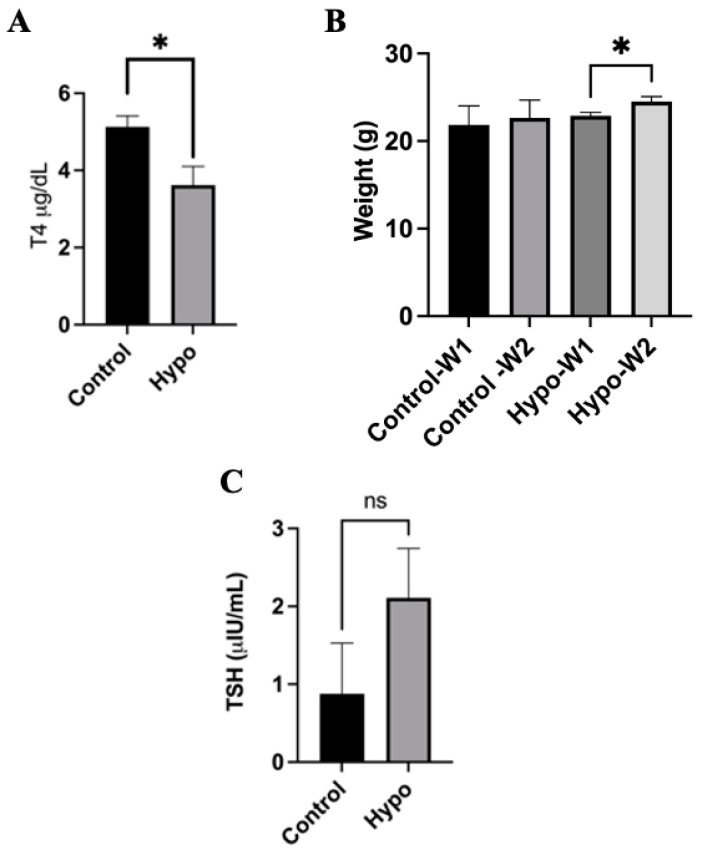
Induction of hypothyroidism in C57BL/6 adult mice. C57BL/6 mice were subjected to a methimazole treatment to induce a decrease in T4 levels. As observed in (**A**), T4 levels were significantly reduced in the methimazole-treated mice (Hypo). (**B**) Increased weight was observed in the Hypo group during the second week of treatment (Hypo-W2); this observation is consistent with what has been observed in hypothyroid patients. Thyroid-stimulating hormone (TSH) levels were also evaluated. (**C**) An increase in plasmatic levels is observed, consistent with a hypothyroid phenotype, but no significant differences were observed. Statistics were carried out by Student’s *t*-test or one-way ANOVA test and Tukey’s post-test; ns: non-significant * *p* > 0.05; *N* = 3 mice per group.

**Figure 2:**
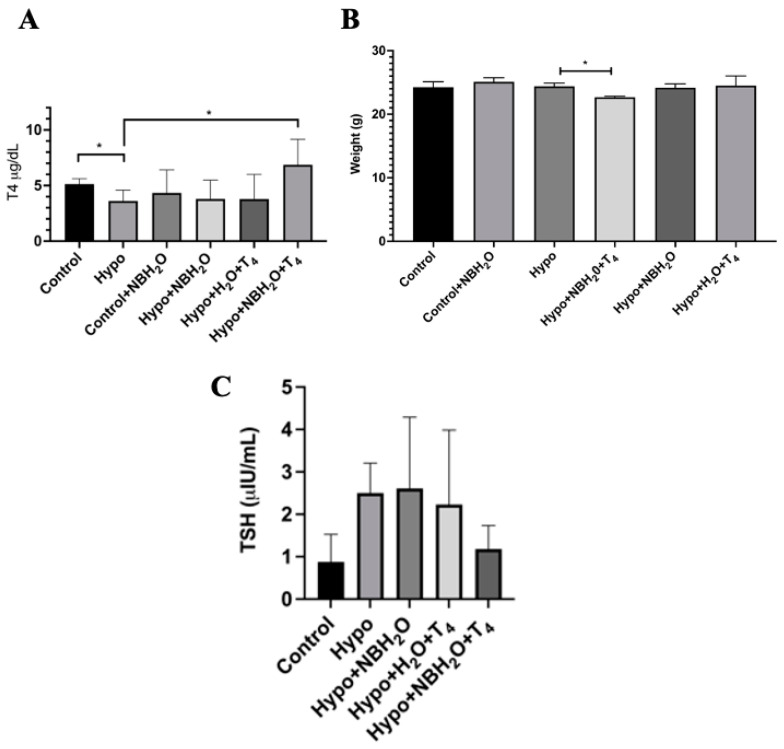
Administration of T4 combined with CO_2_ nanobubbles increases T4 levels in mice plasma. C57BL/6 mice subjected (Hypo) or not (Control) to a methimazole treatment were treated with CO_2_ nanobubbles plus T4 (Hypo + NBH_2_O + T4) or normal water with T4 (Hypo + H_2_O + T4). As observed in (**A**), plasma T4 levels were significantly increased in mice treated with the combination of T4 and CO_2_ nanobubbles (Hypo + NBH_2_O + T4). (**B**) A decrease in weight was observed in the NB+T4-treated group as expected for a hypothyroid phenotype recovery. Thyroid-stimulating hormone (TSH) levels were also evaluated. (**C**) The NB + T4 group presented a decrease in plasmatic levels, but no significant differences were observed. Statistics were carried out by one-way ANOVA test and Tukey’s post-test; * *p* > 0.05; *N* = 3 mice per group.

Transient hypothyroidism was induced in 8-week-old mice. At the end of the treatment, plasmatic levels of T4 and TSH were determined. As observed in Figure 1A, a significant decrease in T4 levels was observed in the Hypo group when compared to the control group. The T4 decrease was accompanied by a small but statistically significant weight increase observed between Hypo animals comparing the first and second week of treatment (Figure 1B), which is consistent with the common hypothyroidism secondary effects reported in human patients [26]. Normally, hypothyroidism is accompanied by an increased plasmatic level of thyroid-stimulating hormone (TSH). Here, we evaluated this parameter and observed an important increase that, however, is not statistically different from the control (Figure 1C). The latter result is likely to be due to the relatively short time frame of the treatment with methimazole and the concentration used to induce a transient T4 decrease [27]. Based on the obtained results, we were able to induce a significant decrease in plasmatic T4 in our animals. Once the decrease in T4 was established in our experimental mice, they were separated into three groups according to the treatment (see Experimental Methods). Results are shown in Figure 2. A significant increase in T4 plasmatic levels was observed in the Hypo group treated with CO_2_-NBs+T4 (Hypo+CO_2_ NBs+T4) compared to the Hypo group. This difference in T4 plasmatic levels was not observed between the Hypo and the normal water+T4 treated group and the group treated with normal or CO_2_-NBs (Figure 2A), thus suggesting that this increase is originated by the introduction of NBs. This is an interesting result given that, in mice, the recovery time for thyroid hormone levels is normally 2 weeks [28], but using the CO_2_-NBs we observed an increase in plasmatic T4 levels already at 5 days after treatment. 

Figure 2B shows a statistically significant weight decrease in the Hypo+CO_2_-NBs+T4 when compared to the Hypo group, consistent with a recovering phenotype. TSH levels were also analyzed, and the results are shown in Figure 2C. No statistically significant differences in TSH levels between all groups were observed, showing that TSH levels in the Hypo+CO_2_-NBs+T4 group were similar to those in the control group. This is consistent with the notion that TSH is not normally a primary marker for thyroid status, and instead the observation of a T4 decrease and body weight gain as thyroid-dependent physiologically effects are more suitable indications for thyroid function alteration [22].

### 2.1. Measurement of CO_2_-NB Size and Zeta Potential

The mean particle size of CO_2_-NBs was found to be 157.1 ± 40.2 nm (PDI: 1.00), and after adding T4, it increased to 282.2 ± 15.7 nm (PDI: 0.66). This increase can be explained by the interaction between T4 molecules and CO_2_-NBs, including the vdW interactions described in the computational analysis. The PDI was employed to gauge nanoparticle stability and formation uniformity. Although initially the CO_2_-NB PDI indicated low uniformity in size distribution, the addition of the T4 molecule improved the PDI, thus enhancing stability and preventing aggregation. Despite the T4-containing NBs exhibiting low monodispersity, their high physical stability and nanometric size (<300 nm) enhance bioavailability and increase the hormone’s biodistribution, highlighting greater treatment efficiency [29]. The zeta potential measurement also was monitored. The zeta potential is the electrostatic potential on the NB surface, affecting physical stability and intermolecular interactions. High zeta potential values prevent aggregation of NBs and increase their stability [30]. The zeta potential values for CO_2_-NBs and CO_2_-NBs+T4 were −9.2 ± 0.32 mV and −8.8 ± 0.9 mV, respectively. Zeta potential is influenced by various factors, particularly the gas type present [31]. The negative surface charge observed on bubble surfaces is primarily attributed to the absorption of OH^−^ ions at the gas–water interface. Each gas exhibits differing abilities that contribute to generating negative charges at these interfaces. The case of CO_2_-NBs presents a notable contrast due to the acidic nature of CO_2_, which readily reacts with water. Upon dissolution, CO_2_ forms the natural bicarbonate system, leading to the generation of carbonic acid through the acid–base equilibrium reaction [32]. Consequently, the resultant low pH environment yields a diminished zeta potential.

Evaluation of CO_2_-NB zeta potential demonstrates a consistent negative charge unaffected significantly by the presence of T4, as corroborated by the existing literature [33,34]. 

The observed low zeta potential values can be attributed to the acidic pH, which intensifies the negative charge at the gas interface. The CO_2_-NBs are more stable at lower pH values, which is the case in an environment such as the mouse stomach. The stomach’s acidic pH may stabilize the CO_2_-NBs until they enter the intestine, allowing the efficient administration of medicinal substances such as T4. The zwitterionic nature of T4 does not substantially influence zeta potential change.

The diameter of nanobubbles was estimated near 282.2 ± 15.7 nm, with a concentration of 10^7^ nanobubbles per mL. The T4 concentration used for in vivo assays was 5 mg in 10 mL, which was divided into doses based on the mouse’s weight. Under these conditions, the theoretical cross-sectional area of the T4 molecule was assumed to be approximately 65 Å^2^, resulting in a T4 concentration per nanobubble of approximately 2.6 M. It is considered that only a small percentage of T4 molecules interact directly with the surface of the nanobubble. In contrast, most T4 molecules probably are organized around the nanobubble, attracted by the organization of surrounding ions and the effect of the electric potential they generate.

### 2.2. Computational Analysis

**Figure 3:**
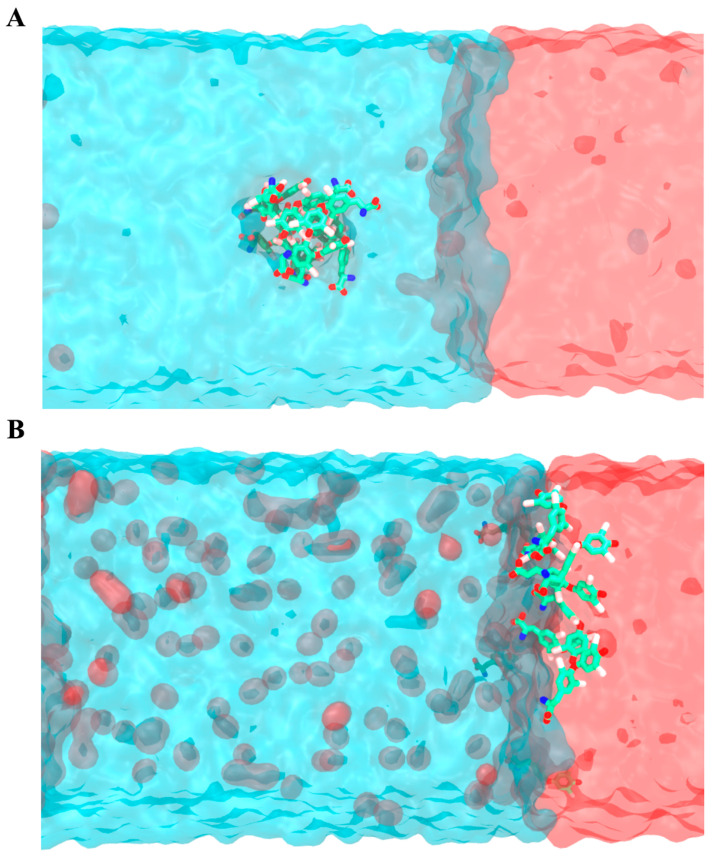
T4 molecules interacting with the water/CO_2_ interface. Initial (**A**) and final (**B**) snapshot (100 ns) depicting the behavior of a T4 (zwitterionic) cluster in water (top) and how the cluster disassembles at the water/CO_2_ interface (**B**). Water molecules appear in cyan, while CO_2_ appears in red. As expected, several CO_2_ molecules escape to the water interface.

With the proposal to evaluate the two protonation states of T4, which are denominated as T4 to represent the neutral state and T4sw to represent the zwitterion protonation state, four unconstrained 100 ns MDs were carried out for each system. Each T4 and T4zw molecule was initially placed in the solvent, away from the water/CO_2_ gas interface, and a molecular cluster of T4zw was built, initially far away from each other. These briefly formed a cluster of T4 molecules. The T4 cluster is a product of the non-bonding vdW interactions that promote the aggregation of organic molecules in the aqueous phase, which was initially formed in a solvated medium and later localized in the solvated phase of the water/CO_2_ system. This type of cluster, commonly generated by drugs, limits the efficiency of the drug and its bioavailability [35]. Naturally, an aggregated drug will have fewer degrees of freedom to interact freely with the receptor, not to mention that the mobility of the drug will be reduced due to its size and could even generate micro-environments that modify the physicochemical properties (e.g., pKa) of the key functional groups for molecular recognition of the drug by the target receptor. Throughout the molecular simulation, the T4 and T4zw molecules spontaneously moved towards and localized at the water/CO_2_ interface, remaining there for the remainder of the simulation until its termination (100 ns) (Figure 3A,B). This process of incorporation at the CO_2_/water interface observed during the MD simulation is complementary with the results previously obtained experimentally. 

Similarly, we observed that the T4 molecular cluster also moves towards the water/CO_2_ interface, where it unbundles. The individual T4 molecules are incorporated into the water/CO_2_ interface of the nanobubble and remain located in this region as individual molecules for the remainder of the simulation. This cluster dissociation process is expected to facilitate the release of the drug as a single molecule, increasing its bioavailability with all the degrees of freedom, as required for an efficient molecular recognition of the drug by its receptor.

**Figure 4:**
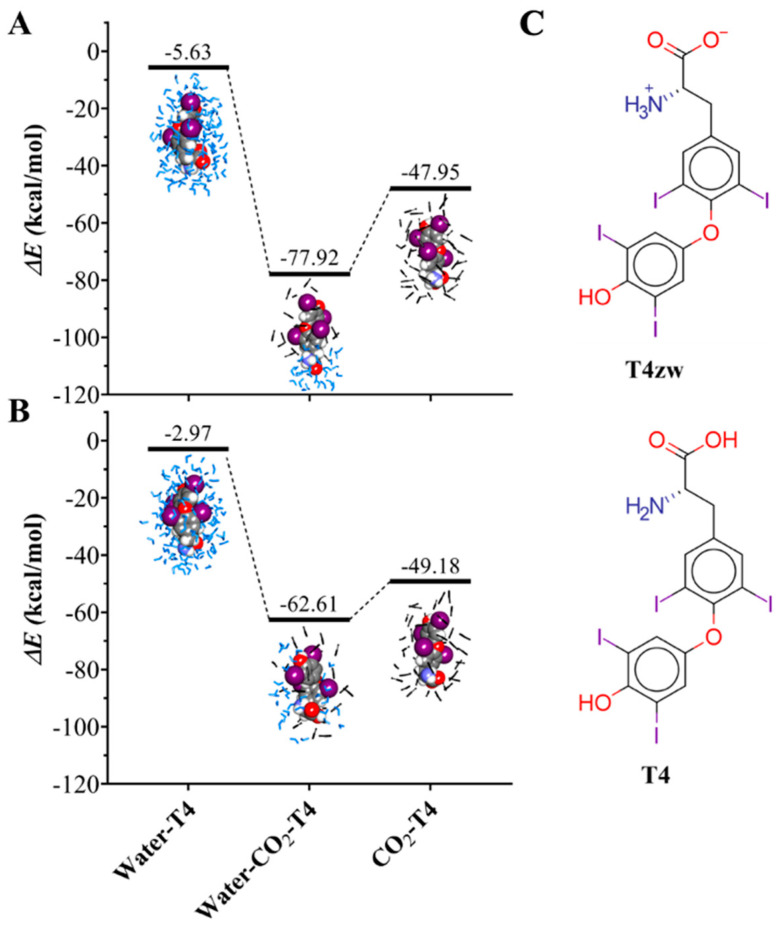
Interaction energy plot. (**A**) T4zw and (**B**) T4 molecules interacting in different phases. CO_2_ in black liquorice representation and water in light blue liquorice representation. (**C**) Chemical structure depiction. Zwitterionic T4 and canonical T4 molecules, highlighted to the C_α_.

To evaluate the energy interactions of T4 with its microenvironment, the interaction energy (∆E) was calculated using the heat of formation (∆H°f) provided by MOPAC at a semi-empirical PM7 level of theory. A clustering analysis was performed, where the most frequent configuration appearing in the molecular simulation was taken for all the complexes, and the results are shown in Figure 4. It can be observed that the T4zw molecule presents a larger (more negative ∆H°f) interaction energy in the three different phases (water, water/gas, and gas), representing a very marked affinity with the water/CO_2_ interface, with a value of −77.9 kcal/mol (Figure 4A). In contrast, the interaction energy of the canonical T4 molecule is significantly higher in the three phases, assuming a value of −62.6 kcal/mol at the interface (Figure 4B). These results imply that T4zw, i.e., T4 in zwitterionic form, tends to locate at the water/CO_2_ interface, contributing to stabilizing the nanobubbles. 

The hydrogen-bond fluctuation plots (Figure S1) and the NCIPLOT isosurfaces (Figure S2) reveal that there are more hydrogen bonds and fewer weak vdW interactions when the T4 molecules are in zwitterionic form (T4zw). These hydrogen bonds are formed between the amino-carboxyl group of T4zw with the water molecules in the liquid phase, while the aromatic rings of T4zw with iodine added at four positions interact with the CO_2_ molecules of the gaseous phase, forming hydrophobic interactions. In the canonical form, T4 has smaller partial charges and hence a smaller dipole moment so that the number of hydrogen bonds decreases, thus favoring a stronger interaction with the CO_2_ gas phase, mainly due to weak vdW interactions. 

Through the representation of these intermolecular interactions, it is possible to analyze the location of T4zw and T4 at the water/CO_2_ system with respect to the water and CO_2_ molecules by means of mass density profile calculations across the water/CO_2_ interface (Figure S3). These profiles show that both the T4 and T4zw molecules are located at the interface, and the canonical and zwitterionic forms are positioned differently according to the intermolecular interactions. In the case of T4zw (Figure S3A), the orientation is mostly perpendicular to the interface, where the (charged) amino-carboxyl group interacts with the aqueous medium and the iodinated rings interact with the CO_2_ gas phase. In contrast, the (canonical) T4 molecule is oriented mostly parallel to the interface, where now the (uncharged) amino-carboxyl group interacts with the two phases. In addition, the hydroxyl group of the terminal aromatic ring of the T4, which forms hydrogen bonds with the solvated medium, favors the parallel orientation.

To further characterize the location and accessibility of T4 molecules at the water/CO_2_ interface, we computed the radial distribution function (RDF) of the distances between T4 molecules and the water/CO_2_ interface (Figure S4). The C_α_ of the amino-carboxyl group of T4zw and T4, the oxygen atom of water, and the carbon atom of CO_2_ were selected as references. The RDF analysis clearly shows that the two molecules present different levels of immersion into the nonpolar CO_2_ gas matrix, with the T4 molecule presenting the greatest immersion and closeness to the nonpolar CO_2_ gas matrix, highlighting the immersion of T4 by about 1.5 nanoseconds into this matrix (Figure 3A). In contrast, the T4zw molecule is found closer to the surface of the nonpolar CO_2_ gas matrix due to the polarity of the amino-carboxyl group and its interaction with the aqueous phase. The RDF characterizing the distance distribution between the C_α_ of the amino-carboxyl group (see Figure S4) and the oxygen atoms of water shows two representative peaks. The more pronounced one is located at approximately (2.9–4.1 Å), while the second one is observed around (4.3–5.6 Å). The height of these peaks in the RDF increases when the T4 molecule is in zwitterionic form and decreases in the canonical T4 form. This is a manifestation of a hydration effect promoted by the hydrogen bonds that form between T4zw and the water molecules, mainly due to the electrostatic attraction in the first solvation layer (Figure S4A). In contrast, the values for the RDF between the C_α_ of the amino-carboxyl group and the carbon atoms of CO_2_ show a representative peak located at approximately (3.4–7.0 Å). In this case, the height of the peaks in the RDF decreases when the T4 molecule is in zwitterionic form and increases for canonical T4, manifesting a nonpolar effect by T4 promoted by the deprotonation of the amino group of canonical T4 (Figure S4B).

### 2.3. Unveiling T4 Translocation across Membranes Treated with CO_2_ Using the Potential of Mean Force Method

**Figure 5:**
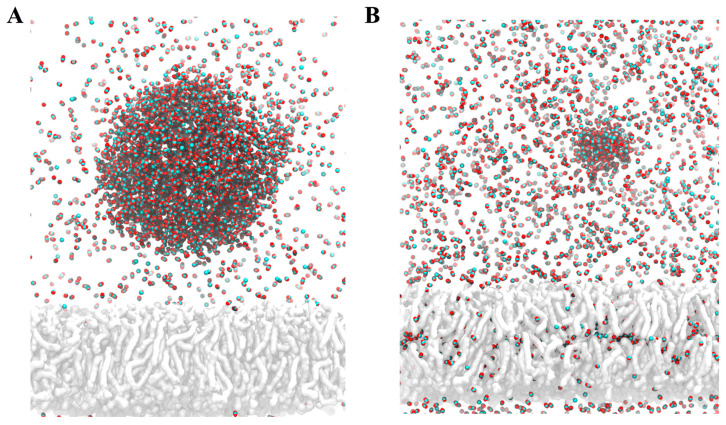
Diffusion of a CO_2_ nanobubble with cell membrane. (**A**) CO_2_ nanobubble placed above the cell membrane at the beginning of the simulation, depicted in red for oxygen atoms and cyan for carbons. Membrane is depicted in grey. (**B**) Snapshot of the last frame of the simulation, showing that CO_2_ molecules from the nanobubbles tend to diffuse towards the center of the membrane.

We simulated the behavior of a CO_2_ nanobubble when it is approaching a cell membrane. Our simulations show that the CO_2_ of the nanobubble rapidly diffuses towards the membrane (Figure 5) and that the gas tends to accumulate in its center, as shown in a density plot in Supplementary Materials. Following these results, we decided to study the permeability of T4 molecules through the CO_2_-filled membrane.

**Figure 6:**
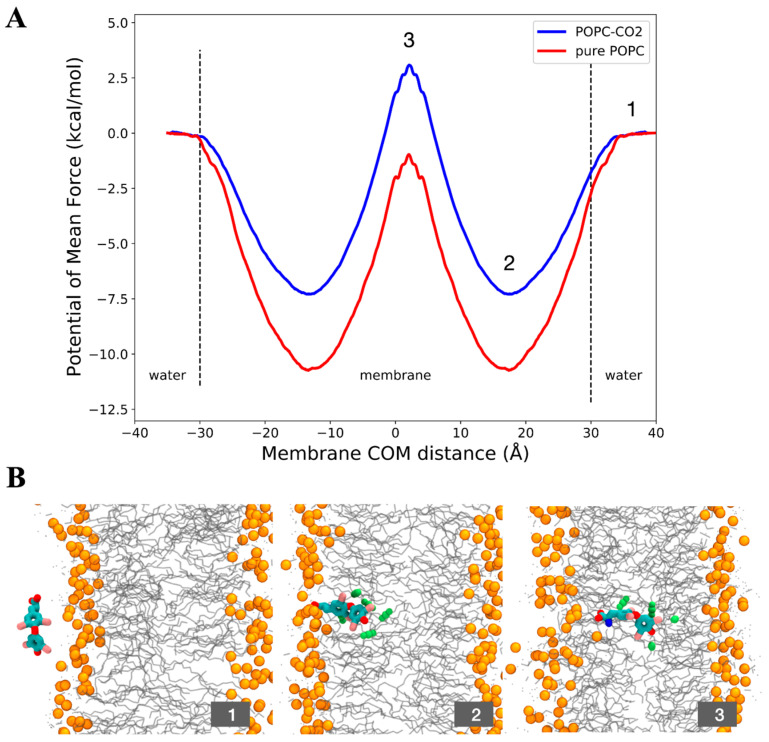
(**A**) Potential of Mean Force of the translocation of a T4 (zwitterionic) molecule through pure POPC and POPC-CO_2_ membranes. (**B**) Snapshots of the conformations of the T4 molecule in different stages of the reaction coordinate: (1) the molecule starts in the water (membrane COM distance = 30 Å), (2) the molecule falls into an energy well in the water–lipid interface (membrane COM distance ~15 Å), (3) T4 is in the middle of the membrane facing a high free energy barrier (membrane COM distance = 0 Å), and T4 falls again into an energy well (membrane COM distance ~−15 Å). Lipid heads appear depicted in orange, in cyan the T4 molecule and in green some CO_2_ molecules around T4.

The potential of mean force (PMF) is a crucial component of the solubility–diffusion model for estimating membrane permeability. It represents the relative solubility of a permeant in solution compared to the membrane interior. According to the conclusions presented above, we decided to inspect the PMF profile only considering T4 in the zwitterionic state, which is the protonation state prevalent in the gas-water interface (see Figure 4). The respective PMFs for T4 through a pure POPC membrane and through a POPC-CO_2_ membrane, depicting their permeation from the water phase to the membrane interior, are presented in Figure 6. The reaction coordinate ξ was chosen as the center of mass distance between T4 and the membrane, projected onto the Z axis.

Examining the profiles, extending from the bulk water region (ξ = −30 Å or ξ = 30 Å) to the polar head group, it appears that the T4 molecule enters easily into both membranes. As the T4 molecule progresses deeper into the bilayer, corresponding to the glycerol region, the molecule exhibits a decrease in free energy in both membranes, reaching deep free energy minima at approximately ξ = 15 Å and ξ = −15 Å, which is the region corresponding to the middle of the outer/inner leaflet of the membrane. In this region, the most hydrophobic part of the molecule is oriented towards the center of the lipids, while the zwitterionic head is oriented towards the polar heads (see Figure 1). However, it should be noted that the minima are lower when the membrane lacks CO_2_ at approximately 4 kcal/mol. This deep energy well in the pure POPC membrane may indicate a longer time of residence of the molecule in this zone, which can decrease the permeation rate through the membrane. 

Then, when the molecule arrives at the center of the bilayer (ξ = 0 Å), it faces an increase in the free energy in both membranes. In the pure POPC membrane this region is purely hydrophobic, while in the POPC-CO_2_ membrane this region is full of CO_2_, which is also energetically unfavorable for T4.

To determine the diffusion of the molecule through both membranes, the self-diffusion coefficient of the T4 molecule was derived from molecular dynamics simulations by fitting the mean squared displacement (MSD) into the Einstein relation. As observed in the Supplementary Materials (Figure S5), the diffusion coefficient of the T4 in a POPC-CO_2_ membrane increases by approximately 4-fold compared to the pure POPC membrane, meaning that the T4 may be retained for some time in the pure POPC membrane before permeating to the inner side, as explained before. Additionally, the order parameter of the POPC lipids were examined in both membranes, showing that POPC-CO_2_ membrane is consistent with a fluid phase state in comparison to pure POPC, which is more similar to a gel phase (not shown).

Additionally, the number of water molecules surrounding the T4 molecule (at 4 Å) as it moves from the water towards the center of the membrane was analyzed (Supplementary Materials Figure S6), in both simulations, with and without CO_2_. The results are in line with those discussed in Figure 4, in which we found that CO_2_ favors interaction with water. As reflected in Figure S6, when the molecule is outside the membrane, it is more surrounded by water molecules compared with the system without CO_2_. This may be related to the fact that the molecule has a certain hydrophobic character and tends to repel water molecules from its closest environment. By adding CO_2_ to the system, and as demonstrated in Figure 4, the interaction of T4 with water is favored.

## 3. Discussion

In this study, we experimentally explored how the use of nanobubbles significantly increases T4 blood concentration in an in vivo assay. To complement this study, we employed molecular dynamics simulation methods and semi-empirical quantum mechanical methods to characterize the structural and energetic properties underpinning the association between the T4 molecule and a CO_2_ nanobubble. Our observations from in vivo assays displayed a 40% increase in blood T4, outperforming other orally administered mixtures. This enhanced blood T4 concentration presents an opportunity to devise new oral administration strategies aimed at reducing drug doses and mitigating side effects arising from drug accumulation. The interaction of T4 with CO_2_ nanobubbles is evidenced in the change in zeta potential and the nanobubble size, which remain relatively consistent. However, a shift in zeta potential is observed due to the association of T4 molecules with the nanobubbles. To scrutinize the interactions steering the association of T4 and CO_2_ nanobubbles, we employed molecular simulation methods. These methods distinctly suggest that T4 molecules preferentially located at the water/CO_2_ interface through the establishment of contacts that equilibrate Van der Waals-type interactions between the gas and the hydrophobic region of T4 and through electrostatic interactions and hydrogen bonds of the polar region of T4 with water molecules at the water/CO_2_ interface. The interactions of T4 with the pure water or gas phases are weaker compared to those observed at the water/CO_2_ interface, implying that T4 acts as a surfactant. In brief, in water and in the water/CO_2_ interface, the T4zw form is more prevalent, with the neutral T4 form being more prevalent in the CO_2_ phase.

Notably, we also discerned that the T4 aggregates spontaneously formed in water are entirely destabilized at the water/CO_2_ interface, allowing individual T4 molecules to freely diffuse over the nanobubble interface. Consequently, the disassembly of T4 clusters augments the bioavailability of this drug and increases the number of degrees of freedom available for molecular recognition by the receptor. The state of the molecule also has an impact on the orientation of the molecule. In the case of T4zw, it orients perpendicular to the interface, while the neutral form tends to be placed parallel to the interface. 

In this study, we have illustrated that the utilization of CO_2_ nanobubbles induces an acidic microenvironment on their surface, facilitating the transformation of the T4 molecule into a zwitterion. This transformation enhances the interaction of CO_2_ nanobubbles with cell membranes, rendering them a highly effective carrier for drugs. This efficacy surpasses that of alternative carriers, such as O_2_-based nanobubbles.

Additionally, the interaction and penetration processes of T4 with the CO_2_ nanobubbles are influenced by the protonation state of T4, whether it is in the zwitterionic (T4zw) or canonical (T4) form. In the interaction at the water/CO_2_ interface, T4zw exhibits a stronger affinity for the water/CO_2_ interface compared to canonical T4, as evidenced by the more negative interaction energy of −77.9 kcal/mol for T4zw versus −62.6 kcal/mol for T4. This stronger affinity is driven by the additive interactions between the hydrophobic region of T4zw and the CO_2_ gas phase, as well as the electrostatic interactions between the polar amino-carboxyl group of T4zw and water molecules at the interface. In contrast, canonical T4, being less polar, interacts more weakly with the interface, primarily through weaker Van der Waals interactions with the CO_2_ phase. The zwitterionic nature of T4zw causes it to orient perpendicular to the water/CO_2_ interface, with the charged amino-carboxyl group interacting with water and the iodinated rings interacting with the CO_2_ gas phase. Conversely, the neutral canonical T4 tends to orient parallel to the interface, with the uncharged amino-carboxyl group interacting with both phases. The radial distribution function (RDF) analysis shows that canonical T4 exhibits greater immersion into the nonpolar CO_2_ gas matrix compared to T4zw, which remains closer to the water/CO_2_ interface due to its polarity. Potential of mean force (PMF) calculations reveal that the presence of CO_2_ molecules in the membrane interior (mimicking a CO_2_ nanobubble-treated membrane) significantly increases the diffusion coefficient of T4zw across the membrane by approximately 4-fold compared to a pure POPC membrane. This enhanced diffusion is attributed to the disruption of the gel-like phase in the pure POPC membrane by the CO_2_ molecules, facilitating the translocation of T4zw. Furthermore, the calculations showed that CO_2_ from nanobubbles improves the permeability of cellular membranes to molecules such as T4, accelerating their diffusion across the membrane. In these calculations, we again observed that T4 has surfactant properties, since the zone of best affinity is the one where its polar groups interact with the membrane heads. 

T4zw exhibits stronger interactions and a perpendicular orientation at the water/CO_2_ interface due to its polarity, while the canonical T4 has a weaker interaction and orients parallel to the interface. Importantly, the presence of CO_2_ molecules (mimicking nanobubbles) significantly enhances the penetration and translocation of T4zw across cell membranes, potentially contributing to the observed increase in T4 absorption in the in vivo experiments. This comprehensive analysis highlights the structure-specific effects of the zwitterionic and canonical forms on the interaction and penetration processes with CO_2_ nanobubbles.

The presence of CO_2_ dramatically increases the diffusion of the drug through the cell membranes, in agreement with our experimental observations that T4 plasmatic levels are increased after 5 days of treatment with CO_2_ nanobubbles instead of two weeks when the T4 was used without nanobubbles. These theoretical and experimental observations contribute to the understanding of the utilization of nanobubbles as efficient drug delivery systems, which could potentially offer pharmacological benefits.

## 4. Materials and Methods

### 4.1. CO_2_-Nanobubble-Supplemented Water 

CO_2_-NB-supplemented water was prepared using a NanoBubble Generator (Holly Technology, Yixing, China) that uses high-pressure rotary flow transient release technology to generate nano-diameter bubbles. Autoclaved tap water and CO_2_ gas were used. NBs were generated for 20 min at 10 °C, using a 4 L/min gas flow. The NB solution was stabilized for 10 min before physicochemical analysis. The NB solution’s pH was determined using a pH meter (model AD1030, Adwa instrument, Szeged, Hungary) with a temperature sensor and a previously calibrated Ag/AgCl reference electrode. Physicochemical properties such as NB size and NB size distribution were determined by Dynamic Light Scattering (DLS). NB stability was evaluated measuring the zeta potential using a Laser Doppler Electrophoresis method. For this purpose, a 1.0 mL aliquot NBs solution was added into a 12 mm polystyrene disposable cuvette (DTS0012). The zeta potential of CO_2_-NBs was measured using a Zetasizer Nano ZS (Malvern Panalytical, Malvern, UK) with a detection angle of 173° and a laser wavelength of 633 nm. Additionally, a 1.0 mL aliquot of NB solution was placed into a disposable folded capillary cell (DTS1070), also for zeta potential determination. Both techniques were performed in triplicate at 25 °C.

### 4.2. Hypothyroidism Induction and CO_2_-Nanobubble Treatment

Eight-week-old C57BL/6 mice were treated with a 0.02% (*w*/*v*) of methimazole (MMI, Sigma, M8506, Milwaukee, WI, USA) in the drinking water for two weeks to induce a decrease in T4 levels. The MMI-treated group was named Hypo. At the same time, a control group was established and defined as mice that only drank normal water during the treatment. After the two weeks of treatment, a blood sample from the facial vein was obtained and T4 and TSH plasmatic levels were determined by Enzyme Immunosorbent Assay (ELISA). The mice’s weight was also registered before and after the 2 weeks of treatment. After MMI treatment, Hypo mice were separated into 3 experimental groups. Hypo treated with normal water (Hypo+H_2_O), Hypo treated with CO_2_-NB-supplemented water (Hypo+CO_2_-NBs), and Hypo treated with CO_2_ nanobubbles and T4 (25 µg/Kg mice weight) supplemented water (Hypo+NBH_2_O+T4). Control mice were separated into two groups, one treated with normal water (Control+H_2_O) and the second group treated with CO_2_-NB-supplemented water (Control+CO_2_-NBs). After five days of treatment, weight was registered, a blood sample was obtained from the facial vein and T4 and TSH levels were determined. All animal procedures were approved by the Ethics committee of the life science faculty at Universidad Andrés Bello. 

### 4.3. Molecular Dynamics (MD) Simulations on the Interaction of T4 with CO_2_

MD simulations were performed for the canonical (T4) and zwitterionic (T4zw) forms of thyroxine in aqueous solutions in the presence of CO_2_ gas. Under physiological conditions, a majority of biomolecules exist predominantly in the zwitterionic form. While the canonical form is also conformationally stable in water, the zwitterionic form is energetically more favorable. The small activation barrier of 4 kcal/mol for the transformation between conformers suggest that there is no kinetic hindrance and hence the partition between canonical and zwitterionic forms is determined by thermodynamic equilibrium [36]. Full geometry optimizations of the T4 and T4zw molecules in the gas phase were carried out using density functional theory with the hybrid exchange correlation functional B3LYP [37] (Becke’s Three Parameter Hybrid DFT-HF exchange functional combined with the LYP correlation functional) in conjunction with the 6–31+G(d) basis set [23]. The force field parameters for the T4 and T4zw molecules were obtained via the LigParGen web server (http://zarbi.chem.yale.edu/ligpargen/, accessed 3 May 2020), which implements the OPLS-AA/1.14*CM1A(-LBCC) force field for organic ligands [38,39,40]. We supplemented this force field with the TIP3P-FB explicit water model [41] and a three-site fully flexible model for CO_2_ [42], re-optimized to better reproduce its vibrational properties [43,44]. The simulations were carried out using the OpenMM software (http://openmm.org, version 8.0.0) [45,46]. Starting configurations were generated in cubic boxes with a lateral dimension of 80 Å. Four systems were prepared for molecular dynamics simulations: (a) two T4 molecules in water/CO_2_ interface, (b) two T4zw molecules in water/CO_2_ interface, (c) ten T4zw molecules in the solvated medium, and (d) a T4zw molecular cluster (formed by ten T4zw molecules) in the presence of a water/CO_2_ interface. To build the water/CO_2_ interface, i.e., the NBs, 6000 water molecules were added on one side of a 120 × 64 × 64 Å^3^ simulation box, and 3400 CO_2_ molecules were added on the other side of the box using the Packmol software 20.14 [47], which generates a starting point for molecular dynamics simulations by packing molecules in defined regions of space. The number of molecules was chosen to obtain a pressure of approximately 30 atm in the CO_2_ region, which corresponds to typical Laplace pressures inside 100 nm radius NBs. A small number of 60 CO_2_ molecules were dissolved in the water phase according to Henry’s law of solubility. First, each system was geometrically optimized for 40,000 steps using the conjugate gradient method and then equilibrated for 1 ns at 300 K in the NVT ensemble. Then, 100 ns long production MD simulations were performed on each system. During the MD simulations, the equations of motion were integrated with a 2 fs time step in the NPT ensemble at a pressure of 1 atm. The SHAKE algorithm was used to constrain bond length for all bonds containing hydrogen atoms, and the Van der Waals (vdW) cut-off distance was set to 12 Å. The temperature was maintained at 300 K by employing the Langevin thermostat method with a relaxation time of 1 ps. A Monte Carlo barostat [39] was used to control the pressure at 1 atm. Long-range electrostatic interactions were computed by means of the particle mesh Ewald (PME) approach. Data were collected every 50 ps during the MD runs. Molecular visualization of the systems and MD trajectory analysis were carried out with the VMD software package (version 1.9.4) [48]. 

### 4.4. Intermolecular Interaction Energy

The strategy for calculating the intermolecular interaction energy of T4 and T4zw in water and CO_2_ phases is as follows: each fragment of the system was considered separately, these fragments being the T4 molecules, *water/CO_2_*, and the complex between T4 and *water/CO_2_*, each of which was optimized separately using the PM7 semi-empirical quantum-mechanical method implemented in MOPAC16 [49]. Subsequently, the heat of formation (Δ*H_f_*) was calculated for each fragment using PM7. Finally, the intermolecular interaction energy (Δ*E*) was obtained according to the following Equation (1):

(1) $$∆E={∆H{}^{\circ}f}_{complex}-({∆H{}^{\circ}f}_{T4}+{∆H{}^{\circ}f}_{water/{CO}_{2}})$$

### 4.5. Noncovalent Interaction Index (NCI)

To reveal possible non-covalent T4 and T4zw interactions in the presence of the CO_2_ solvated interactions, such as hydrogen bonds, steric repulsion, and vdW interactions, we used the non-covalent interaction index (NCI) [50]. The NCI is based on the electron density (*ρ*), its derivatives, and the reduced density gradient (*s*). In this work, the level of theory used was the PM7 semi-empirical method of MOPAC16 [49]. The NCI was calculated using the GPUAM software (https://github.com/gpuam/binaries) (accessed on 1 April 2024) [51].

### 4.6. Molecular Dynamics Simulations on the Nanobubble–Membrane Interaction and Potential of Mean Force Method

To study the nanobubble-membrane interaction, a POPC membrane patch, 160 × 160 Å2, was placed into a box of 160 × 160 × 198 Å^3^, composed of 128,316 water molecules. A nanobubble of radius = 100 Å was generated, composed of 4415 CO_2_ molecules. The system was submitted to 5000 minimization cycles, with positional restraints in the CO_2_ molecules and POPC phosphate groups of 10 kcal/mol × Å^2^. Restraints were progressively eliminated. Then, a production stage was performed at 310 K using a Monte Carlo barostat for 100 ns. 

The potential of mean force (PMF) profiles for the T4 molecule translocating across pure-POPC and POPC-CO_2_ membranes were determined using the umbrella sampling method [44]. A 110 × 110 × 80 Å^3^ membrane patch, obtained from the nanobubble–membrane simulation, composed of 248 lipids was constructed, and an extra 20 Å of water was added to both sides of the *z*-axis. The molecule was placed on the upper side of each of the two systems and restrained using a harmonic restraint of 10 kcal/mol × Å^2^ during a 100 ns equilibration simulation. To simulate the translocation of the molecule across the membrane, the reaction coordinate ξ for the umbrella sampling method was defined as the distance between the center of mass of the molecule and the center of mass of the membrane, projected onto the *z*-axis. The reaction coordinate was divided into 70 windows of 1 Å. A harmonic spring with a force constant of 1 kcal/mol × Å^2^ was applied to each window. At least 20 ns of simulation were performed for each window, resulting in a total simulation time of approximately 1400 ns for each system. The PMF profiles were then calculated using the weighted histogram analysis method (WHAM) [52]. The lipids were modeled using the CHARMM36 force field [53]. The T4 molecule was parameterized using the CHARMM General force field (CGenFF) [54,55]. The reference temperature and pressure were set to 310 K and 1 atm, respectively. The time step was fixed at 2 fs. Temperature control was achieved using the Nose–Hoover thermostat, and pressure control was maintained using a semi-isotropic pressure scheme. Temperature and pressure time constants of 0.1 and 1.0 ps, respectively, were employed. Long-range Coulomb interactions were computed using the particle mesh Ewald (PME) method. A cut-off distance of 1.4 nm was used for both Van der Waals and Coulomb interactions. All simulations were carried out using the AMBER 22 package, http://ambermd.org (accessed on 1 April 2024) [56]. 

### 4.7. Statistical Analysis

The data were analyzed using Student’s *t*-test or Mann–Whitney U test when two experimental groups were compared, and one-way ANOVA test and Tukey’s post-test were used when three or more groups were analyzed. The GraphPad Prism 8.0.2 program was used for statistical analysis. The results were considered significantly different when *p* < 0.05.

## 5. Conclusions

The in vivo experiments showed that administering thyroxine (T4) combined with CO_2_ nanobubbles to hypothyroid mice led to a significant 40% increase in blood T4 levels compared to administering T4 alone in normal water. This enhanced absorption of T4 with CO_2_ nanobubbles was observed after just 5 days of treatment, whereas normally it takes 2 weeks for T4 levels to recover without nanobubbles. The increased T4 absorption with nanobubbles was accompanied by a decrease in body weight in the hypothyroid mice, consistent with reversal of the hypothyroid phenotype. 

Molecular dynamics simulations revealed that T4 molecules spontaneously localize at the water/CO_2_ interface of the nanobubbles, driven by a combination of hydrophobic interactions between T4 and the CO_2_ gas phase and electrostatic interactions between the polar groups of T4 and water. At the water/CO_2_ interface, clusters of aggregated T4 molecules were observed to dissociate into individual molecules, increasing bioavailability. The zwitterionic form of T4 exhibited stronger binding affinity for the water/CO_2_ interface compared to the neutral canonical form. Potential of mean force calculations showed that the presence of CO_2_ molecules in the membrane interior, mimicking a nanobubble-treated membrane, significantly increased the diffusion and translocation of T4 across the membrane by around 4-fold compared to a normal membrane. 

The CO_2_ nanobubbles markedly enhanced absorption and bioavailability of the thyroid drug T4 in vivo, and computational modeling provided molecular insights into how the nanobubbles facilitate T4 binding at the gas–water interface and penetration into cell membranes.

## Data Availability

Data are contained within the article or Supplementary Materials.

## Supplementary Materials

The following supporting information can be downloaded at: https://www.mdpi.com/article/10.3390/ijms25115827/s1.
