# Supplementary material for: Increased Absorption of Thyroxine in a Murine Model of Hypothyroidism Using Water/CO2 Nanobubbles
Source: Int J Mol Sci. 2024 May 27;25(11):5827. doi: 10.3390/ijms25115827 (PMC11172067; doi:10.3390/ijms25115827)
Supplement: Supplementary file 1 [file ijms-25-05827-s001.zip › ijms-2986354-supplementary.pdf]

Supplementary Material

Increased absorption of thyroxine in a murine model of hypothyroidism using water CO_2 nanobubbles

Ma. Cecilia Opazo[2,3*], Osvaldo Yañez[2], Valeria Marquez-Miranda[1], Johana Santos[2], Maximiliano Rojas[1], Ingrid Araya-Durán[1], Daniel Aguayo[1,4], Matías Leal[1], Yorley Duarte[1,4], Jorge Kohanoff[5,6] and Fernando D. Gonzalez-Nilo[1,4*]

[1]Center for Bioinformatics and Integrative Biology (CBIB), Facultad de Ciencias de la Vida, Universidad Andres Bello, 8370146, Santiago, Chile.
[2]Facultad de Medicina Veterinaria y Agronomía, Instituto de Ciencias Naturales, Universidad de las Américas, Santiago, Chile
[3]Millenium Institute on Immunology and Immunotherapy, Laboratorio de Endocrino Inmunología, Facultad de Ciencias de la Vida, Universidad Andrés Bello.
[4]Interdisciplinary Center for Neuroscience of Valparaíso, Faculty of Science, University of Valparaíso, 2340000, Valparaíso, Chile
[5]Instituto de Fusión Nuclear "Guillermo Velarde", Universidad Politécnica de Madrid, Madrid 28006, Spain
[6]Atomistic Simulation Centre, Queen's University Belfast, Belfast BT7 1NN, United Kingdom

*Correspondence:
Dr. Ma. Cecilia Opazo. Instituto de Ciencias Naturales, Facultad de Medicina Veterinaria y Agronomía, Universidad de las Américas, Santiago, Chile
Email: mopazod@udla.cl

Dr. Fernando Danilo Gonzalez-Nilo. Center for Bioinformatics and Integrative Biology (CBIB), Facultad de Ciencias de la Vida, Universidad Andres Bello, 8370146, Santiago, Chile
Email: fernando.gonzalez@unab.cl

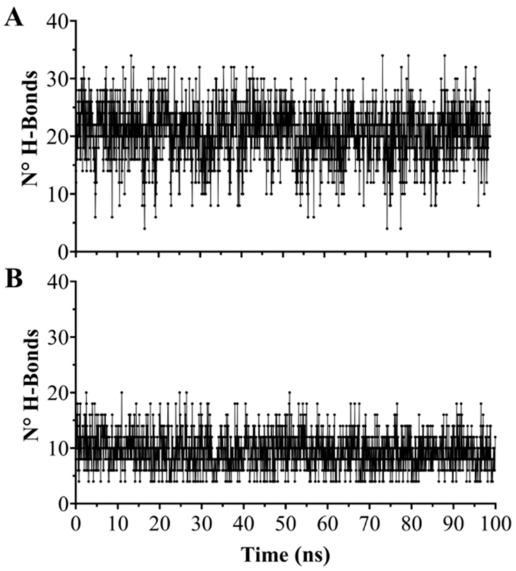

Figure S1. Hydrogen bonds as a function of simulation time for A) T4zw and B) T4 molecules.

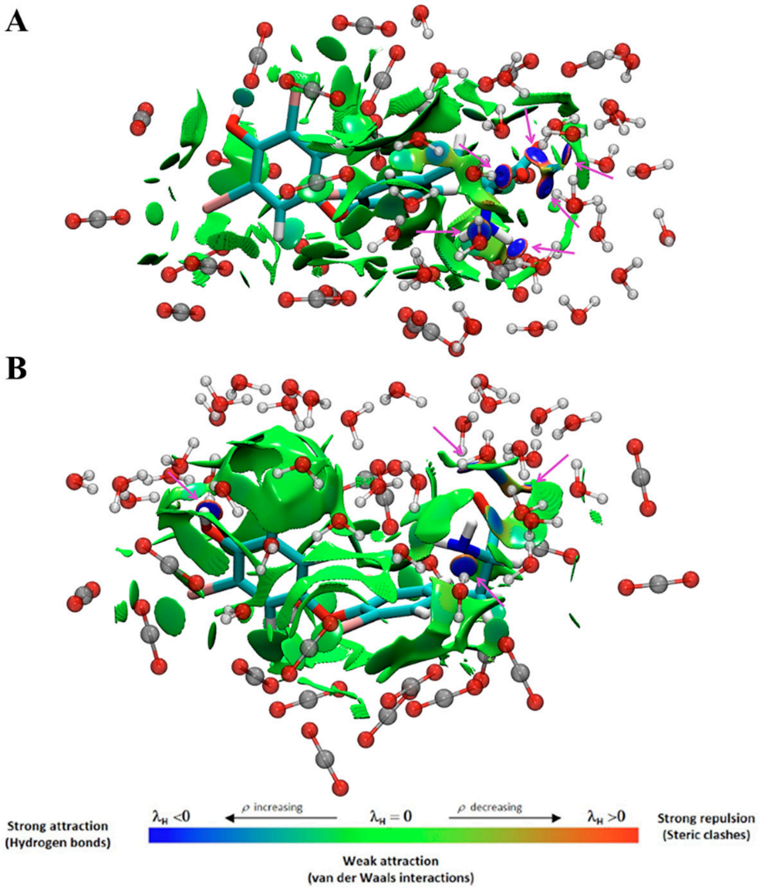

Figure S2. NCIPlot depicting intermolecular interactions. Non-covalent interactions isosurface gradient (0.6 au) between A) T4zw and B) T4 molecules on mixed water/CO_2. The surfaces were coloured on a blue-green-red scale according to the strength and type of interaction. Blue indicates strong attractive interactions, green indicates

weak Van der Waals interactions, and red indicates a strong non-bonded overlap. The arrows indicate possible interactions between T4 molecules and the water/CO_2 interface.

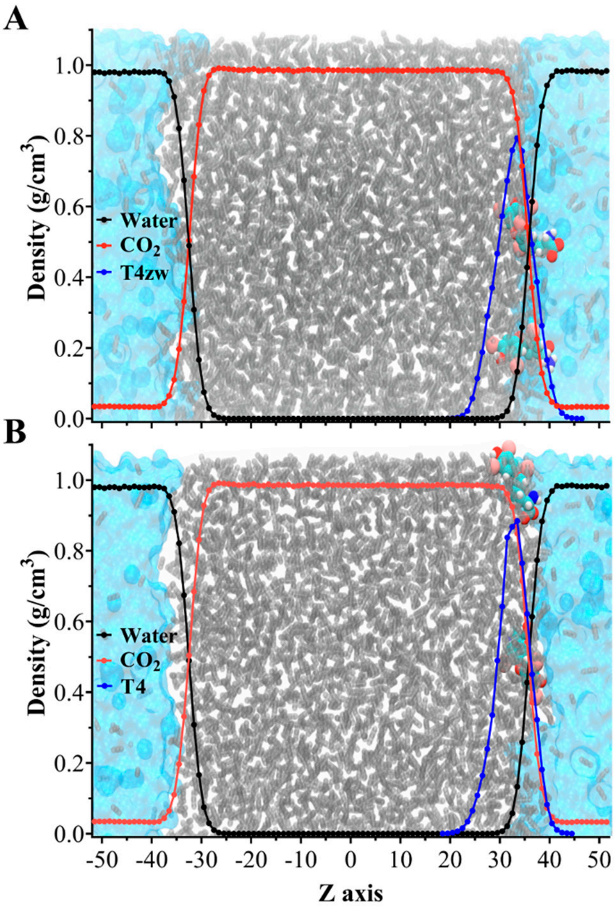

Figure S3: Mass density profile. Component-wise mass density profiles for systems with A) T4zw and B) T4 in water/CO_2 interface.

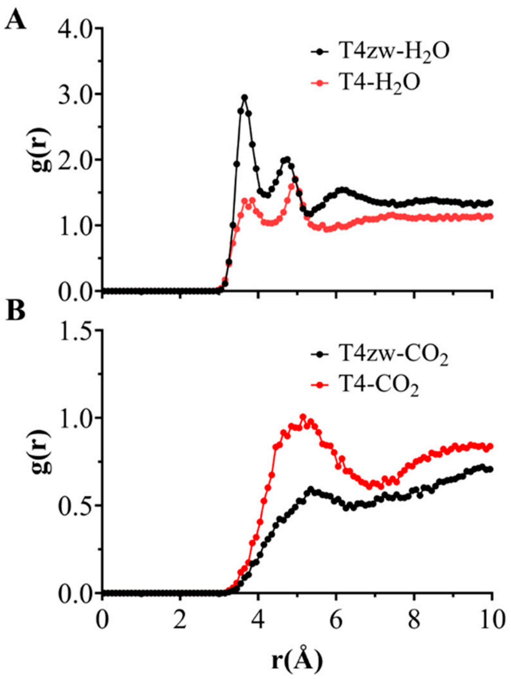

Figure S4. Average radial pair distribution functions (RDF) for A) oxygen from the water and B) carbon from the CO_2 against alpha carbon of the amino group of T4zw and T4.

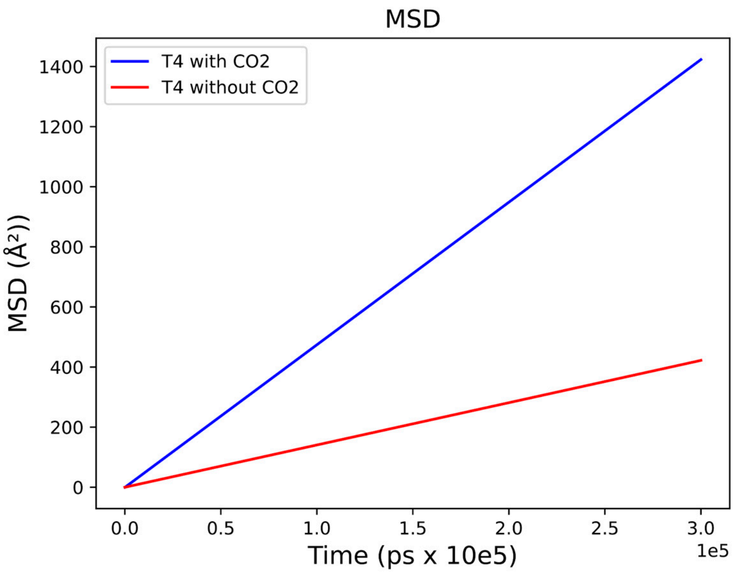

Figure S5. Mean Square Displacement (MSD) of the T4 molecule through cell membranes considering a pure-POPC membrane (red) and a POPC-CO2 membrane (blue), obtained from molecular dynamics simulations.

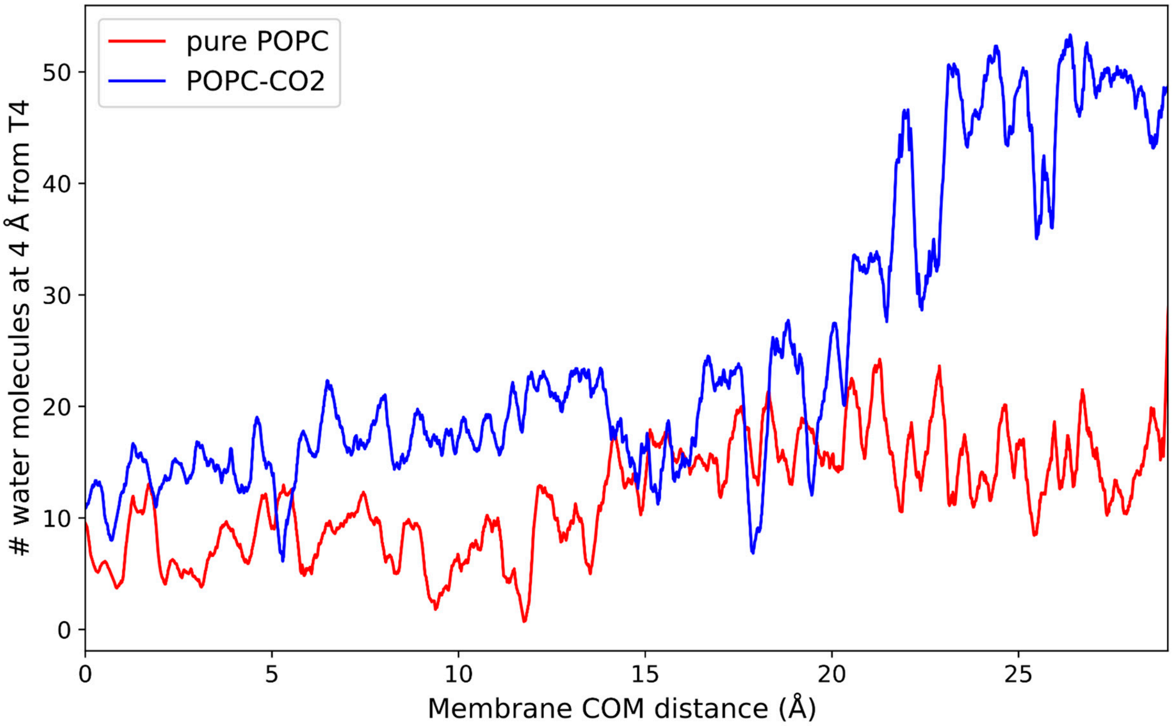

Figure S6. Number of water molecules at 4 surrounding the T4 molecule in both simulations (pure-POPC and POPC-CO_2). The center of the membrane is in x=0, while x=30 represents the extracellular side.
